# RAD51C and MYST3 Mutations in a Case of Desmoid-Type Fibromatosis With No Mutation in CTNNB1 or APC

**DOI:** 10.7759/cureus.55496

**Published:** 2024-03-04

**Authors:** Keith M Skubitz, Paari Murugan

**Affiliations:** 1 Medicine, University of Minnesota, Minneapolis, USA; 2 Laboratory Medicine and Pathology, University of Minnesota, MInneapolis, USA

**Keywords:** sarcoma, beta-catenin, apc, ctnnb1, myst3, rad51c, aggressive fibromatosis, fibromatosis, desmoid

## Abstract

Most cases of desmoid-type fibromatosis (DTF) exhibit a mutation in APC or CTNNB1. We report a case of mesenteric DTF in which no mutation in APC or CTNNB1 was found, but a germline variant of uncertain significance (VUS) in RAD51C and a subclonal mutation in MYST3 were identified. Whether these genetic changes are important in DTF in this case, or whether genetically conventional DTF cells were present at a density below detection is unknown; it will be of interest to see results in further studies of wild-type APC/CTNNB1 cases.

## Introduction

Desmoid-type fibromatosis (DTF), also known as aggressive fibromatosis or desmoid tumor is a monoclonal proliferation of myofibroblast-like cells that has a variable course and does not metastasize but can be locally aggressive [1,2]. The Wnt/beta-catenin pathway plays an important role in DTF biology, and DTF is much more common in patients with germline APC mutations [1-3]. Sporadic DTF usually has a mutation in CTNNB1, the gene that encodes beta-catenin. Rare cases of DTF have been reported that lack an identified mutation in CTNNB1 or APC [4-7] and cases with more than one mutation in CTNNB1 have been reported (reviewed in [8]).

A model of DTF pathogenesis involves an activation stimulus in the setting of dysregulation of the Wnt/beta-catenin pathway resulting in beta-catenin upregulation, nuclear translocation, and increased Wnt target gene expression resulting in cell proliferation and extracellular matrix (ECM) production [2,3,8]. Other signaling molecules associated with this process may also recruit normal non-clonal myofibroblast precursor cells such as prophylactic ADAM12 positive cells from a PDGFR-positive precursor pool, thus adding normal non-clonal myofibroblasts to the true DTF myofibroblasts [2,4].

We report a case of mesenteric DTF in which no mutation in APC or CTNNB1 was found, but a germline variant of uncertain significance (VUS) in RAD51C and a sub-clonal mutation in MYST3 were identified. Whether these genetic changes are important in DTF in this case, or whether genetically conventional DTF cells were present at a density below detection is unknown; it will be of interest to see results in further studies of wild-type APC/CTNNB1 cases.

## Case presentation

**Figure 1:**
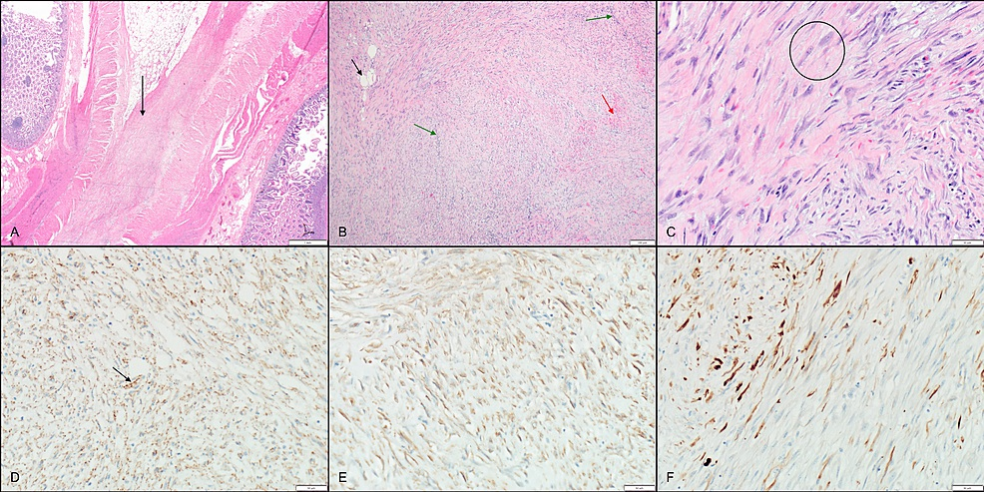
Histological sections of tumor. Aggressive fibromatosis infiltrating mesenteric adipose tissue (arrow) resulting in adhesion between intestinal segments (left and right) (H&E x40) (A). Typical architectural features of desmoid-type fibromatosis including intersecting cellular fascicles of fibroblasts with adipocytic entrapment (black arrow), compressed slit-like blood vessels (green arrows) and extravasated red blood cells (red arrow) (H&E x100) (B). Characteristic myofibroblastic cytology in the tumor cells, including plump spindled appearance with amphophilic cytoplasm and elongated oval nuclei containing multiple small nucleoli (circle) (H&E x400) (C). Beta catenin immunohistochemical stain demonstrating cytoplasmic reactivity comparable to vascular endothelium (arrow) with the absence of nuclear reactivity (IHC x200) (D). Smooth muscle actin immunohistochemistry showing diffuse reactivity in the tumor cells, confirming myofibroblastic differentiation (IHC x200) (E). Patchy immunoreactivity for desmin in the tumor cells, uncommonly encountered in typical cases of aggressive fibromatosis (IHC x200) (F).

A 41-year-old man presented with abdominal pain and had a laparoscopic lysis of adhesions. He had an appendectomy in the distant past and had been felt to have irritable bowel syndrome. A month later, he had bowel obstruction with CT imaging showing wall thickening of several loops of ileum and low-grade small bowel obstruction. He had a bowel resection at which time a mesenteric desmoid tumor was removed along with a section of bowel. Several plaque-like areas that were felt to be fibromatosis were noted, but there was no attempt to surgically remove these. Histological sections demonstrated sweeping fascicles of moderately cellular spindle cells infiltrating the mesenteric adipose tissue and involving the serosal aspect of the intestinal wall. The spindle cells demonstrated plump fusiform nuclei, small multiple nucleoli, and amphophilic cytoplasm in a collagenized and focally myxoid background. Scattered inflammatory cells, extravasated red blood cells, and slit-like blood vessels with perivascular edema were also present. There was no evidence of increased nuclear atypia, tumor necrosis, or mitotic activity. An immunohistochemical (IHC) stain showed the tumor was positive for smooth muscle actin. Desmin demonstrated patchy reactivity while beta-catenin showed cytoplasmic reactivity (similar to background connective tissue) without nuclear staining. CD34 was negative (Figures 1A-1F). These morphologic features were characteristic of DTF despite the absence of convincing beta-catenin immunoreactivity. Other entities including reactive myofibroblastic proliferation, idiopathic fibrosis, smooth muscle neoplasm, gastrointestinal stromal tumor, low-grade fibromyxoid sarcoma, dedifferentiated liposarcoma, and myofibroblastic sarcoma were considered in the differential diagnosis. However, the morphologic features best fit a DTF diagnosis. The tumor was diagnosed as DTF at the Mayo Clinic; immunohistochemical stains for beta-catenin did not show convincing nuclear reactivity. Consultation at the University of Minnesota (PM) confirmed the diagnosis of mesenteric DTF as well (Figures 1A-1F). Colonoscopy did not reveal any polyps. 

His past history is notable for appendectomy, obesity, umbilical hernia, exercise-induced asthma, and ocular migraines. His family history is notable for no history of colon polyps but cancer in multiple family members (Table 1).

**Table 1:** Family history Family members who developed malignancy.

Relation	Malignancy
Father	melanoma
Paternal grandfather	Renal cell cancer
Paternal grandmother	Renal cell cancer
Paternal uncle	Lung cancer
Paternal cousin	leukemia
Maternal grandfather	melanoma
Maternal grandmother	Renal cell cancer leukemia
Paternal uncle	unknown cancer
Paternal uncle #2	unknown cancer
Maternal aunt	Uterine cancer
Maternal uncle	Renal cell cancer Colon cancer

Due to his family history, his primary physician referred him to a genetics clinic where the germline status of 80 cancer-related genes using the Invitae Multi-Cancer Panel platform, revealed, a variant mutation in RAD51C, exon 3, c.523G>T (p.Ala175Ser) heterozygous mutation. CTNNB1 mutation testing was done at the OHSU Knight diagnostic laboratories, Portland, OR. The tumor was examined microscopically to identify a suitable area, macro-dissected, and DNA extracted and purified. The estimated tumor cellularity in the tested material was 80%. Exon 3 of CTNNB1 was amplified by PCR and the products screened for mutations by bidirectional Sanger sequencing with an estimated sensitivity of 20% mutant allele. No CTNNB1 mutation was detected.

Next-generation sequencing (NGS) of the tumor using the FoundationOne platform found a sub-clonal MYST3 4222G>A (E1408K) mutation. Several variants of uncertain significance (VUS) (CAD (H1577Q), EED (K284R), FGF14 (K3fs*74), FLT3 (D909H), IKBKE (E351K), KDR (L462V), MCL1 (L144P), NOTCH2 (L2408H), NUP98 (P1063L), TNFRSF11A (D427N)) were also identified but no CTNNB1 or APC mutation was identified. On the last follow-up he is doing well with no evidence of recurrence 8.8 years after resection.

## Discussion

Dysregulation of the Wnt/beta-catenin signaling system is felt to be important in DTF biology, and almost all cases have either a mutation in CTNNB1 or APC, although rare cases have been reported with no mutation in either gene [4-7]. Koike et al. described IHC staining for beta-catenin in 104 cases of DTF and found 84% showed nuclear staining and 91% showed staining of the cytoplasm [5]. Crago et al. reported the results of a comprehensive study that examined molecular aberrations in 16 cases of “wild-type” DTF. They showed that the wild-type tumors were predominantly located in the abdominal wall and had a similar rate of negative nuclear beta-catenin staining to CTNNB1 mutant tumors. Although whole exome and deep sequencing detected CTNNB1/APC/Wnt pathway mutations in a majority of the wild-type samples tested, three had no somatic mutations that could be linked to Wnt/beta-catenin signaling [4]. The current case also had no mutation in CTNNB1 or APC but had mutations in two other genes not previously reported in association with DTF.

A heterozygous germline mutation was found in the RAD51C gene, exon 3, c.523G>T (p.Ala175Ser). The sequence change replaces alanine with serine at codon 175 of the RAD51C protein; the alanine residue is highly conserved and there is a moderate physiochemical difference between alanine and serine. Algorithms developed to predict the effect of missense changes on protein structure and function do not agree on the potential impact of this missense change (SIFT: “tolerated,” PolyPhen-2: “possibly damaging,” Align-GVGD: “class C0”). This variant is not present in population databases (ExAc no frequency) and has not been reported in the literature in individuals with a RAD51C-related disease. This mutation is thus classified as a VUS. Mutations that affect the function of the RAD51C gene have been linked to a moderately increased risk for breast and ovarian cancer in people who carry a single pathogenic RAD51C variant [9-11], and there is also at least one case report of childhood Fanconi anemia-like disorder in the setting of biallelic RAD51C mutations (one from each parent) [12]. However, there is currently insufficient data to determine if this particular variant in RAD51C causes cancer/reproductive risk or simply represents a benign individual variant. His family history is positive for cancer. It is unclear if this VUS contributed to the DTF.

NGS of the tumor also found a sub-clonal MYST3 E1408K mutation. MYST3 (also known as KAT6A) encodes a histone lysine acetyltransferase known as MOZ. The t(8:16)(p11;p13) rearrangement associated with M4 and M5 subtypes of acute myeloid leukemia involves a fusion of MYST3/MOZ on chromosome 16 and the CREB-binding protein locus CBP [13,14]. Somatic sequence alterations, primarily missense substitutions in MYST3 have been found at low frequency in a range of solid tumors [15], but the functional impact of these alterations remains to be defined.

Either or both of these genetic changes could contribute to the biology of DTF in this case. Finally, it is possible that these genetic findings are unrelated to the DTF and the number of true tumor cells in the DTF may be too low for detection with the majority of the tumor cells representing normal myofibroblasts recruited to the tumor by undefined growth factors. Further genetic testing in DTF cases with no detectable APC or CTNNB1 mutation is warranted. In addition, it is of interest to determine the proportions of mutant and normal myofibroblasts in DTF tumors and examine correlations with natural history and response to treatment.

## Conclusions

This case describes a patient with DTF lacking identifiable mutations in CTNNB1 or APC, but a germline mutation in RAD51C of uncertain significance and a sub-clonal mutation in MYST3. Either or both of these genetic changes could contribute to the biology of DTF in this case and further genetic testing in DTF cases with wild-type APC and CTNNB1 is warranted. Alternatively, it is possible that these genetic findings are unrelated to the DTF and the number of true tumor DTF cells in the tumor may be too low for detection with the majority of the tumor cells representing normal myofibroblasts recruited to the tumor by undefined growth factors.
